# Biochemical Characterization, Antifungal Activity, and Relative Gene Expression of Two *Mentha* Essential Oils Controlling *Fusarium oxysporum*, the Causal Agent of *Lycopersicon esculentum* Root Rot

**DOI:** 10.3390/plants11020189

**Published:** 2022-01-11

**Authors:** Seham A. Soliman, Elsayed E. Hafez, Abdu M. G. Al-Kolaibe, El-Sayed S. Abdel Razik, Sawsan Abd-Ellatif, Amira A. Ibrahim, Sanaa S. A. Kabeil, Hazem S. Elshafie

**Affiliations:** 1 Plant Protection and Biomolecular Diagnosis Department, Arid Lands Cultivation Research Institute, City of Scientific Research and Technology Applications, Borg EL-Arab, Alexandria 21934, Egypt; sehamsoliman50@yahoo.com (S.A.S.); elsayed_hafez@yahoo.com (E.E.H.); eshabaan@srtacity.sci.eg (E.-S.S.A.R.); amiranasreldeen@yahoo.com (A.A.I.); 2 Microbiology Department, Faculty of Science, Taiz University, Taiz 6803, Yemen; abdu_71@yahoo.com; 3 Bioprocess Development Department, Genetic Engineering and Biotechnology Research Institute, City of Scientific Research and Technology Applications, Borg EL-Arab, Alexandria 21934, Egypt; sabdellatif@srtacity.sci.eg; 4 Protein Research Department, Genetic Engineering and Biotechnology Research Institute, City of Scientific Research and Technology Applications, Borg EL-Arab, Alexandria 21934, Egypt; sanaa.protein@gmail.com; 5 School of Agricultural, Forestry, Food and Environmental Sciences, University of Basilicata, Viale dell’Ateneo Lucano 10, 85100 Potenza, Italy

**Keywords:** WRKY transcription factor, essential oils, Fusarium root rot, *Mentha spicata*, *Mentha longifolia* GC–MS, antioxidant enzymes, antifungal activity

## Abstract

Tomato (*Lycopersicon esculentum* Mill.) is important food in daily human diets. Root rot disease by *Fusarium oxysporum* caused huge losses in tomato quality and yield annually. The extensive use of synthetic and chemical fungicides has environmental risks and health problems. Recent studies have pointed out the use of medicinal plant essential oils (EOs) and extracts for controlling fungal diseases. In the current research, *Mentha spicata* and *Mentha longifolia* EOs were used in different concentrations to control *F. oxysporum*. Many active compounds are present in these two EOs such as: thymol, adapic acid, menthol and menthyl acetate. These compounds possess antifungal effect through malformation and degradation of the fungal cell wall. The relative expression levels of distinctly upregulated defense-related WRKY genes (WRKY1, WRKY4, WRKY33 and WRKY53) in seedling root were evaluated as a plant-specific transcription factor (TF) group in different response pathways of abiotic stress. Results showed significant expression levels of WRKY, WRKY53, WRKY33, WRKY1 and WRKY4 genes. An upregulation was observed in defense-related genes such as chitinase and defensin in roots by application EOs under pathogen condition. In conclusion, *M. spicata* and *M. longifolia* EOs can be used effectively to control this plant pathogen as sustainable and eco-friendly botanical fungicides.

## 1. Introduction

The tomato plant (*Lycopersicon esculentum* Mill.) is considered one of the third most important vegetable plants worldwide. Moreover, it is one of the most widespread vegetable crops grown across the globe. The tomato plant is highly sensitive to various biotic and abiotic stresses, which results in high economic losses [1,2]. The biotic stress which affects tomato plant growth and production is *Fusarium oxysporum* f. sp. *lycopersici* (*Fol*) [3]. *Fusarium oxysporum* is a soil-borne pathogen that targets the plant by attacking the tomato roots, resulting in wilt disease [4]. Wilt disease frequency in tomato crops is very high in some countries where it reaches up to 25 ± 5% [5,6].

Additionally, in the presence of suitable conditions for the fungus, especially in developing countries, economic losses may increase up to 80% [7]. Consequently, the fungus gains its capability in tomato infection through secretion of mycotoxins [8], which have hazardous effects on animal and human health [9]. Mint essential oil (EO) has been reported to have a strong antimicrobial activity against several pathogenic microorganisms [10,11]. Many researchers have studied the biological activity of different EOs from Mentha against different pathogenic fungi, especially *Fusarium* species [11,12,13,14,15]. In particular, the two studied *Mentha* EOs have illustrated a strong antifungal activity against potato pathogens in addition to different soil-borne diseases in tomatoes [16,17,18].

The most active chemical compounds in EOs of *Mentha* species are piperitone oxide, pulegone, and 3-cyclopenten-1-one, 2-hydroxy-3-(3-methyl-2-butenyl) [19,20]. These compounds play an important role in defense against pests, pathogens, and fungi [21,22,23]. Sharma et al. [24] studied the effect of mint, clove, lemongrass, and eucalyptus EOs on wilt-causing fungus *F. oxysporum.* Plants’ EOs have a vital role in enhancing plant defense systems by increasing the production of phytochemicals as phenolic compounds and peroxidases enzymes which lead to strengthening of the cell wall and increasing lignification against phytopathogens [25].

When any pathogen infects plants, it is well known that they induce a plant’s defense system which works to resist both the pathogen attack and development of disease [26]. The plant defense system works once the plant is exposed to any stress; plant transcription factors belonging to multiple families play a critical role in stress mitigation or other adjustment mechanisms by modulating the gene expression patterns [27]. There is a large gene family, “WRKY”, which is considered the transcriptional factors distributed in all plant parts [28]. In addition, the WRKY genes were previously discovered in non-photosynthetic eukaryotes [29], and consequently have been identified and characterized in different plant species [30,31].

The main role of WRKY genes is defense; these genes work in the plant acquired resistance by using different pathways, including different enzymes [32]. Researchers have reported that they play a role in the defense mechanism of the *Arabidopsis* plant infected with necrotrophic fungal pathogens *Botrytis cinerea* and *Alternaria brassicicola* [33]. Several studies revealed that WRKY genes might bind with the promoter of phytoalexin deficient 3 and 1-aminocyclopropane-1-carboxylic acid synthase 2 when the plant is attacked by *Botrytis cinerea* [34].

The aims of the present study are (i) investigating the potential antifungal activities of different concentrations of *M. spicata* and *M. longifolia* EOs against Fusarium root rot disease caused by *F. oxysporum* in tomato plant; (ii) demonstrating the possible alterations in seedling germination, total phenols, and the activity of several antioxidant enzymes; and (iii) discovering the mode of action between the fungus and plant through analyzing the expression levels of defense-related genes as chitinase (PR3) and defensin (PR12) and *WRKY* transcriptional factors (TFs) (such as WRKY1, WRKY4, WRKY33, and WRKY53) by investigating the upregulation or downregulation profile of studied defense and WRKY genes against *Fusarium* attack.

## 2. Results

### 2.1. Screening of Antifungal Activity of Studied EOs

**Figure 1:**
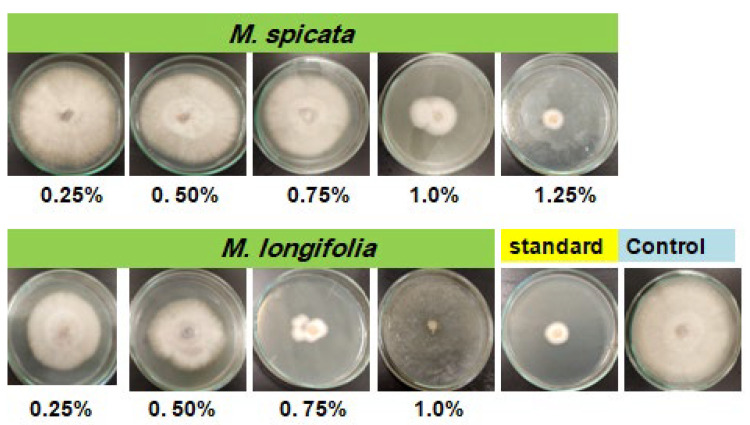
Effects of the two studied EOs at different concentrations on mycelial growth reduction percentages of *F. oxysporum* after 7 days of incubation at 28 ± 2 °C.

Antifungal activity of *M. spicata* and *M. longifolia* EOs at various concentrations (0.25%, 0.5%, 0.75%, 1.0%, and 1.25%) was investigated against *F. oxysporum*. The average reductions in *F. oxysporum* radial growth in response to *M. spicata* and *M. longifolia* EOs colloid treatment are shown in the graph (Figure 1).

All tested concentrations exhibited varied inhibitory activity compared with positive control fungicide (nystatin 0.005%) and the untreated experimental control. Inhibition of mycelium growth increased with time and complete growth reduction was achieved after 7 days of incubation at 28 ± 2 °C in the case of *M. longifolia* EOs at 1.0% and 1.25%. Whereas, the highest significant growth inhibition (92.55** ± 0.08% and 90.63** ± 0.04) was achieved in the case of *M. spicata* EO (1.25%) and nystatin (0.005%), respectively, compared with control. The lowest insignificant growth inhibition, 14.33 ns%, was obtained with *M. spicata* EO at 0.25%.

### 2.2. Chemical Composition of Mentha EOs

**Figure 2:**
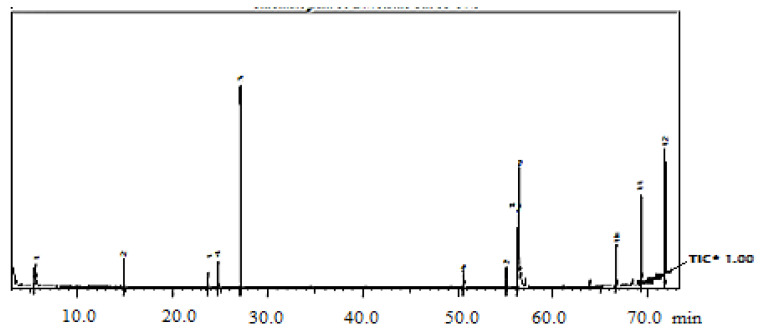
GC–MS chromatogram of *M. spicata* EO. Where (*) is the Total Ion Current.

**Figure 3:**
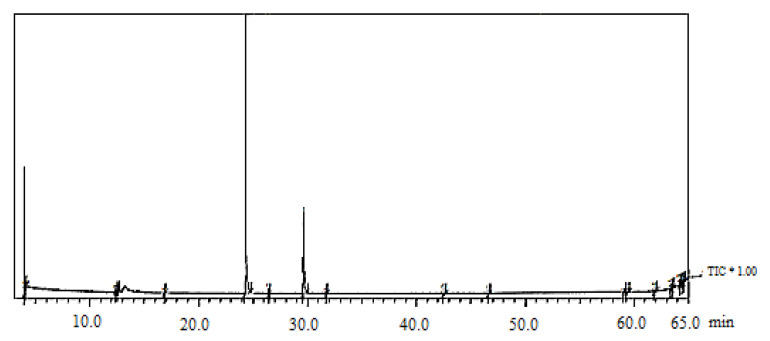
GC–MS chromatogram of *M. longifolia* EO. Where (*) is the Total Ion Current.

Selected GC–MS analysis chromatograms of the two studied EOs are shown in Figure 2 and Figure 3. The component relative concentrations (calculated as GC peak area percentages and retention index) are shown in Table 1 and Table 2. GC–MS results identified that the principal bioactive components belonged to different chemical groups. The major constituents in *M. spicata* EO included thymol (28.19%), adipic acid (25.82%), piperitone (24.76%), and menthol (24.18%), whereas, the major constituents in *M. longifolia* EO included menthol (51.4%), menthyl acetate (20.5%), and d-limonene (11.15%).

### 2.3. Effects of EOs on Tomato Growth Parameters

**Figure 4:**
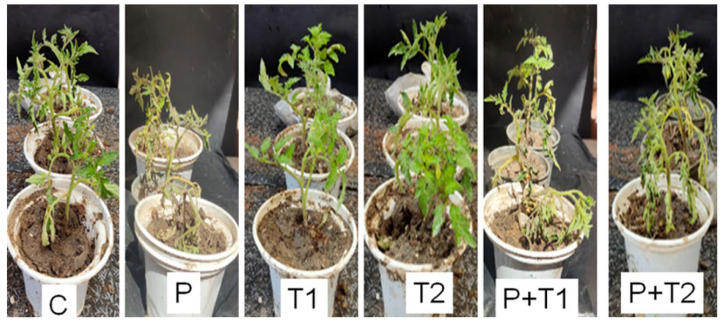
Effects of highest concentrations of *M. spicata* and *M. longifolia* EOs formulations on *L. esculentum* seedling growth potential under Fusarium root rot disease infection. C = control, P = pathogen, T1 = 1.25% *M. spicata*, T2 = 1% *M. longifolia*, P + T1 = pathogen + 1.25% *M. spicata*, and P + T2 = pathogen + 1% *M. longifolia*.

Tomato seedlings, treated with the highest concentrations (1.0% and 1.25%) of *M. spicata* and *M. longifolia*, respectively showed in vitro complete and maximum reduction in mycelial growth in *F. oxysporum* colonies and disease incidence of Fusarium root rot compared with untreated control (Figure 4). The magnitude of root rot symptoms and disease severity (86.39 ± 0.025) was observed in *Fusarium* inoculated experiments, whereas a significant reduction in root rot disease severity by 5.6 ± 0.01% and 3.5 ±.02% was shown in treated plants with 1.25% *M. spicata* and 1.0% *M. longifolia* EOs treatments.

**Figure 5:**
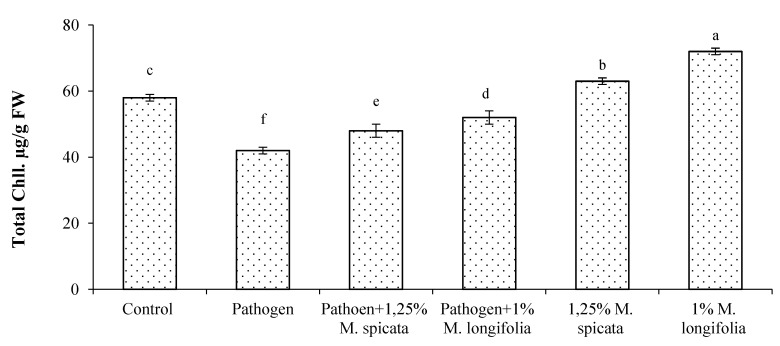
Effects of highest concentrations of *M. spicata* and *M. longifolia* EOs on total chlorophyll content (Chll) in *L. esculentum* seedling under Fusarium root rot disease infection. Bars with different letters indicate significant differences between treatments at *p* ≤ 0.05. Data are expressed as the mean of three replicates ± SDs.

**Figure 6:**
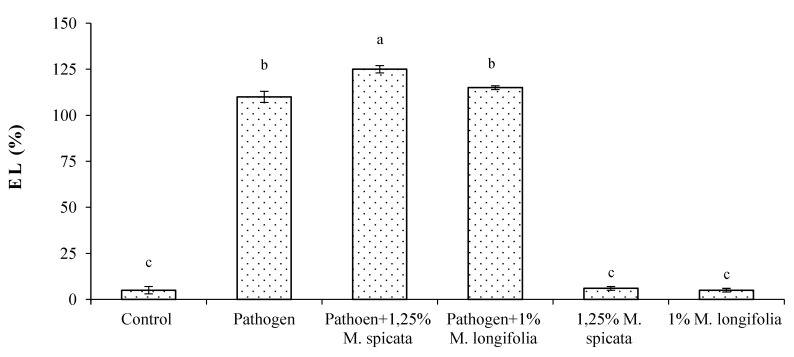
Effects of highest concentrations of *M. spicata* and *M. longifolia* EOs on electrical leakage percentage (EL%) in *L. esculentum* seedling leaves under Fusarium root rot disease conditions. Bars with different letters indicate significant differences between treatments at *p* ≤ 0.05. Data are expressed as the mean of three replicates ± SDs.

In addition, the application of 1.25% *M. spicata* and 1.0% *M. longifolia* EOs exhibited a significant increase in the following growth parameters: plant height, shoot and root fresh, and dry weights, compared with treated-infected, infected, and negative control EOs (Table 3). A remarkable maximum plant height (32.42 ± 0.02 cm) was observed in the case of 1.0% *M. longifolia*-treated plants followed by (28.9 ± 0.01 cm) with 1.25% *M. spicata* treatment compared with control (24.32 ± 0.02). In contrast, the lowest plant height (16.54 ± 0.02) was observed in the case of pathogen-inoculated plants. The treatment with 1.0% *M. spicata* and *M. longifolia* resulted in a significant increase in all other measured germination features such as radicle and root lengths, and fresh and dry weights of radicle and root compared with control (Figure 4), while germination features were greatly decreased due to fungal infestation, where a clear root rot severity was observed. The total chlorophyll content and electrical leakage results are shown in Figure 5 and Figure 6 where the maximum chlorophyll content (74.16 ± 0.01 and 62.33 ± 0.02) were measured in 1.0% *M. longifolia* and 1.25% *M. spicata*, respectively, compared with *Fusarium* inoculated plants (42.06 ± 0.02). Whereas, the maximum electrical leakage (EL %) (127.18 ± 0.01) was recorded in pathogen-treated plants with 1.25% *M. spicata* compared with (6.97 ± 0.01) in case of control.

### 2.4. Protein, Total Phenols, Flavonoids, Malondialdehyde Contents, and Antioxidant Enzymes

**Figure 7:**
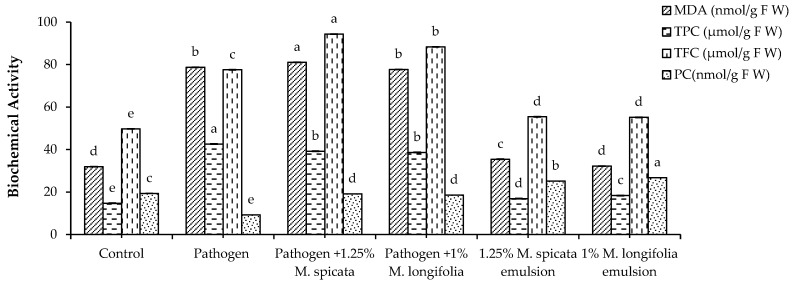
Effects of highest concentrations of *M. spicata* and *M. longifolia* EOs on MDA, TPC, TFC, and PC level in *L. esculentum* seedling under Fusarium root rot disease infection. Bars with different letters indicate significant differences between treatments at *p* ≤ 0.05. Data are expressed as the mean of three replicates ± SDs.

**Figure 8:**
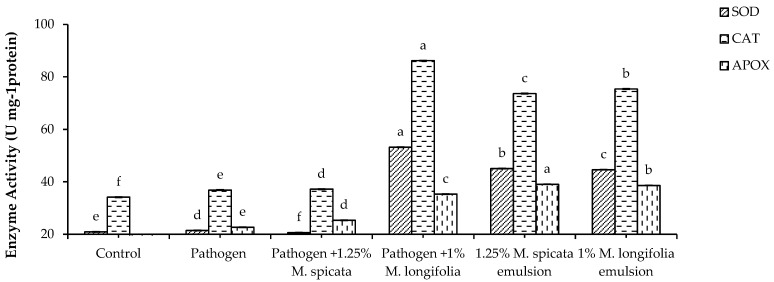
Effects of *M. spicata* and *M. longifolia* EOs on SOD, CAT, and APOX activity in *L. esculentum* seedling under Fusarium root rot disease infection. Bars with different letters indicate significant differences between treatments at *p* ≤ 0.05. Data are expressed as the mean of three replicates ± SDs.

Data presented in Figure 7 and Figure 8 represent the effects of *M. spicata* and *M. longifolia* application on *L. esculentum* seedling on lipid peroxidation level, protein content (PC), total phenols content (TPC), total flavonoids content (TFC), and different antioxidant enzymes of *L. esculentum* seedling in all treatments of 35 DAS and 1.25% *M. spicata* and 1.0% *M. longifolia* application under Fusarium root rot infection. Overall, the pathogen-infected plants showed significant increase in MDA level, TPC, and TFC contents compared with the untreated control, whereas *M. spicata* and *M. longifolia* applications showed a slight increase in MDA, TPC, and TFC. In addition, PC of pathogen-infected plants treated with 1.25% *M. spicata* and 1.0% *M. longifolia* had the highest content at 19.09 µmol/g and 18.54 µmol/g of FW, respectively, compared with control and pathogen-infected plants.

Assessed results of antioxidant enzymes in Figure 8 show the effect of *Fusarium* infection and 1.25% *M. spicata* and 1.0% *M. longifolia* applications for two weeks of seedling transplantation on SOD, CAT, and APOX enzymes activities in *L. esculentum* plant leaves. Our results reveal a significant increase in SOD, CAT, and APOX enzymes activities in all treatments and *Fusarium*-infected experiments compared with untreated control.

### 2.5. qRT-PCR of the Plant Defence System

qRT-PCR was carried out with mRNA to assess the expression levels of different WRKY transcription factors WRKY1, WRKY4, WRKY33, and WRKY53 (Figures S1–S4) which play an important role in biotic and abiotic tolerance. In addition, qRT-PCR represented the relative expression levels of defense-related proteins such as chitinase (PR3) and defensin (PR12) genes (Figures S5 and S6) in tomato plant roots after two weeks from *Fusarium* inoculation and EOs application, respectively.

**Figure 9:**
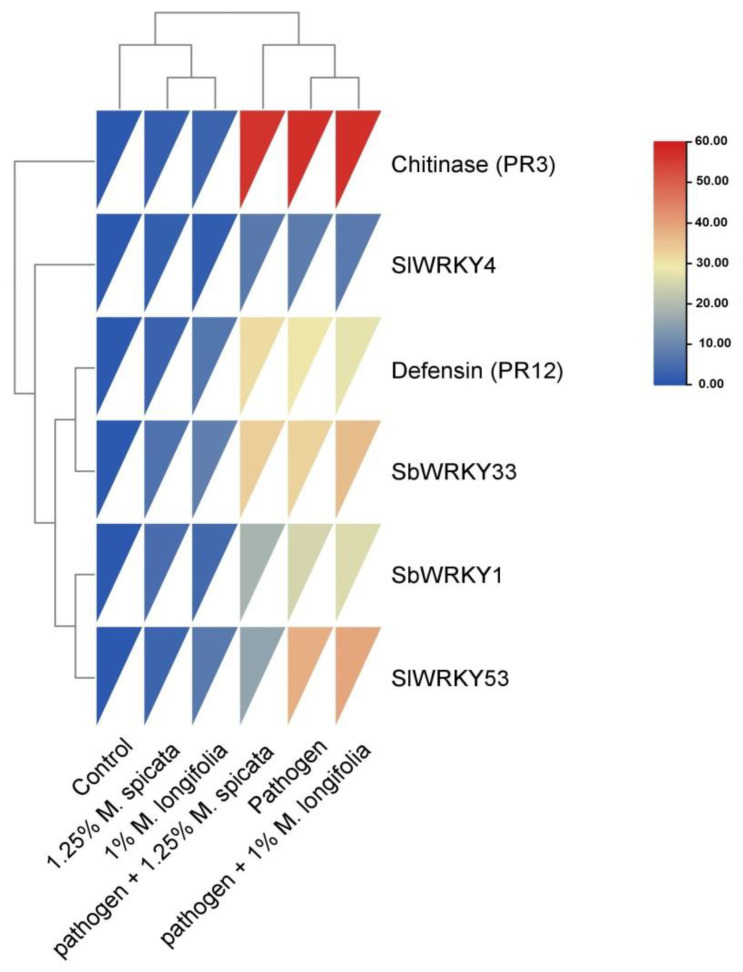
Hierarchical clustering heat map of relative expression levels of chitinase, defensin genes, and WRKY1, WRKY4, WRKY33, and WRKY53 transcripts in *L. esculentum* seedling under Fusarium injection and application of 1.25% *M. spicata and* 1.0% *M. longifolia* EOs treatments.

In our study, the highest expression mRNA level (57.24 ± 0.01) was recorded for chitinase gene in *L. esculentum* plant roots at 1.0% *M. longifolia* under Fusarium infection, followed with (57.16 ± 0.02, 56.4 ± 0.02) of pathogen and *M. spicata* application treatments, respectively, compared with (1.0 ± 0.0) in untreated control treatments. Overall, the WRKY TFs genes (WRKY1, WRKY4, WRKY33, and WRKY53) in tomato seedling roots showed positive expression levels (upregulation) under *M. spicata* and *M. longifolia* treatments and positive control, compared with the untreated control. The highest expression mRNA levels of WRKY transcriptional factors WRKY53 gene (39.233 ± 0.03) represented in tomato roots at 1.0% *M. longifolia* treatment under Fusarium infection condition, followed with (38.12 ± 0.02) in pathogen treatment. Figure 9 shows a hierarchical clustering heat map and the correlation among different treatments and their gene expression. The red color demonstrates the highest correlation, and the blue color demonstrates the lowest correlation.

## 3. Discussion

*F. oxysporum* f. sp. *radicis-lycopersici* is a wide-spread fungus in the plant rhizosphere which causes Fusarium crown and root rot (FCRR) disease and leads to losses of tomato production even in greenhouses and systems of soil production [35]. There are different management methods for root rot disease of tomato crop using chemical and biological controls [36]. Biological control for pathogenic fungi is the new management trend for reducing the harmful effects of chemicals (fungicides) [37,38]. There are four types of biocontrol management: microorganisms, semi-chemical products, plant-based natural products, or living microorganisms [39,40,41,42,43]. Furthermore, plant EOs are effective biocontrol agents against a variety of pathogenic fungi and bacteria [44,45,46,47].

The present investigation revealed the effect of *Mentha spicata* and *M. longifolia* on EOs on root rot disease of tomato infected with *F. oxysporum* both in vitro and in vivo. *M. spicata* and *M. longifolia* EOs had potentiality against *Fusarium*. The order of efficient EOs against *Fusarium* pathogen was: *M. longifolia* > *M. spicata*. The highest antifungal activity was observed in the case of all concentrations of *M. longifolia* EO in agreement with previous studies [48]. The capability of two *Mentha* EOs against fusarium is due to the ability of bioactive chemical molecules to penetrate the fungal cell wall and cytoplasmic membrane and destroy mitochondrial membranes [49]. Plant EOs contributed to loss of rigidity of the hyphal cell wall as well as damaging the cellular enzyme system, resulting in cell death [50,51]. Many other studies reported that the antifungal activity of *M. spicata* EO against *F. oxysporum* and *Aspergillus niger* depends on the chemical constituents: menthol, thymol, and piperitone, individually or in synergic effect [52,53]. Thymol compound activity presented in the malformation of the cellular membrane in addition to inhibition for ATPase activity [43,54]; regarding the effect of thymol and eugenol, they correlated to the ability of thymol compounds’ lysis of the external membrane of microorganisms which facilitated the entrance of eugenol to cytoplasm and interacted with protein [44,53]. Krishna Kishore et al. [55] demonstrated the antifungal activity of carvacrol, -terpineol, terpinen-4-ol, and linalool against *Rhizoctonia solani*, *F*. *oxysporum*, *Penicillium digitatum*, *A. niger*, *Alternaria alternate*, and *A. flavus*; these produce an effect against different microbial cells due to the ability of these compounds to penetrate the cell membrane, inactivate the enzyme pathway, and disturb their active transport [56,57].

The biological effect of the studied EOs on physiological parameters of tomato seedling is due to the presence of terpenes, alcohols, and phenolic compounds [55,58,59,60]. Moreover, the infected plants treated with *M. longifolia* EO showed the highest values of plant height (19.86 cm), shoot fresh weight (13.64 g), shoot dry weight (1.85 g), root fresh weight (1.41 g), and root dry weight (0.14 g). The main components in *M. spicata*, adipic acid 25.82% and piperitone 24.76%, are different than their percentage in *M. spicata* EO as reported by Bayan and Küsek [61]. Chemical constituents for *M. longifolia* EO were d-limonene, menthol, menthyl acetate, linalool, and eugenol, with percentages that differ from those reported from GC–MS analysis conducted by Desam et al. [62]. This difference in the percentage of single constituents for *Mentha* EOs may be due to differences in extraction methods or genetic diversity of these plants [63].

Menthol, menthyl acetate, linalool, and eugenol constituents alter cell permeability for *Fusarium* fungi and cause plasmolysis and cell death [64]. This study recorded collapsing of mycelium hyphae for *F. oxysporum* f. sp. *lycopersici* treated with EOs of *M. spicata and M. longifolia* with potential activity as biological control and therapeutic effect against root rot disease.

To analyze the role of chitinase, defensin, WRKY1, WRKY4, WRKY33, and WRKY53 transcripts in *L. esculentum* plant defense against *F. oxysporum* fungal pathogen, we analyzed their expression after pathogen infection, and pathogen infection and application of 1.25% *M. spicata and* 1.0% *M. longifolia* EOs treatments. The expression results showed over-expression by 57.24 fold and changes in the level of WRKY33 transcription factor were recorded in infected plants treated with 1.0% *M. longfolia*, followed with 57.16 and 56.4 fold changes in pathogen, and 1.25% *M. spicata* treatments, respectively, while minimum expression of WRKY33 2.43 fold was observed with 1.25% *M. spicata* EO compared with control. The expression profile of WRKY35 TF revealed a significant upregulation of 39.23 and 38.12 fold changes at the pathogen-infected plant under 1.0% *M. longfolia* and pathogen treatments, respectively, and the minimum fold change of 3.52 was at 1.25% *M. spicata* EO compared with control. Data obtained in this study showed upregulation in the WRKY4 TF expression of 37.65 and 32.95 change folds at the pathogen-infected plant under 1.0% *M. longfolia* and pathogen treatments, respectively. In comparison, WRKY1 TF expression patterns were 26.35 and 25.17 change folds at the pathogen-infected plant under 1.0% *M. longfolia* and pathogen treatments, respectively, as compared with minimum expression level of 4.23 folds with 1.0% *M. longfolia*. Our results were in agreement with previous studies which reported that *WRKY3* and *WRKY4* encode two structurally similar WRKY proteins, and their expression was responsive to stress conditions. Stress-induced expression of *WRKY4* but not *WRKY3* was further enhanced by pathogen infection. These results strongly suggest that WRKY4 regulates crosstalk between SA and JA/ET-mediated signaling pathways and, as a result, plays opposite roles in resistance to the two different types of microbial pathogens. Interestingly, WRKY proteins such as WRKY4, WRKY33, and redundant WRKY18, WRKY40, and WRKY60 play a positive role in plant resistance to necrotrophic pathogens.

The expression of defense-related genes showed over-expression under pathogen infection conditions and with the pathogen under 1.0% of *M. longfolia* and 1.25% of *M. spicata*. In contrast, chitinase gene was upregulated with 31.25, 29.6, and 27.83 fold changes at pathogen with 1.25% *M. spicata*, pathogenated plants, and then pathogenated with 1.0% *M. longfolia* treatments, respectively. In addition, defensin gene expressed as 18.65, 16.16, and 15.76 fold changes at pathogen with 1.0% *M. longfolia*, pathogen with 1.25% *M. spicata*, and pathogen treatments compared with minimum 2.44 fold expression was recorded at 1.25% *M. spicata* EO treatment.

These results collectively indicate that overexpression of chitinase, defensin, and WRKY transcripts play a positive role in inducing plant resistance against *F. oxysporum* and working toward reducing disease severity. Additionally, WRKY transcripts and PR3, PR12 genes were further enhanced by pathogen infection, and they are already considered as a marker for the plant–microbe interaction.

WRKY family members have diverse regulatory mechanisms; their protein can be effectively combined with W-box elements and bind to acting elements to activate or inhibit the transcription of downstream target genes through the cis-acting mechanism [65]. Thus, WRKY as a transcription factor plays an important role in plant defense in response to attacks by several pathogens. The response works by activating the expression of resistance genes directly or indirectly. It has been reported that WRKY DNA binding proteins bind to the promoter region of Arabidopsis natriuretic peptide receptor 1 (NPR1), which activated the plant defense system [66]. Moreover, WRKY33 activates the plant resistance system against necrotrophic fungi *Alternaria brassicicola* and *Botrytis cinerea* [29], and it can regulate the SAR system in the infected plants and also the PR genes [67,68]. Moreover, the high expression of such transcription factors could regulate the plant pathogen sensitivity to mutants of AtWRKY4, AtWRKY3, and AtWRKY3 WRKY4, increasing the plant susceptibility toward the fungus *B. cinerea*. In contrast, the high expression of the non-mutated AtWRKY4 enhanced the plant’s resistance toward the *Pseudomonas syringae* [69]. Many plant WRKY genes are induced by biotrophic and necrotrophic pathogens, including fungi and viruses, through the induction of SA-dependent SAR and PR genes [70,71].

## 4. Materials and Methods

### 4.1. Sample Collection, Identification, and Preparation

#### 4.1.1. Fungal Isolate and Tomato Variety

The studied *Fusarium oxysporum* isolate was investigated as an aggressive fungal pathogen in tomato and other plants and was deposited in GenBank under accession number (KJ831189) and obtained from the Department of Plant Protection and Biomolecular Diagnosis, Arid Lands Cultivation Research Institute, Alexandria (Egypt). The fungal isolate was maintained on potato dextrose agar (PDA) slants and stored at 4 °C until further bioassay. Tomato seed (super strain B) variety was obtained from the Egyptian Ministry of Agriculture.

#### 4.1.2. Medicinal Plant Materials

Leaves of *M. spicata* and *M. longifolia* were collected from unrestricted habitats, Alexandria (Egypt), in June 2020. The plants’ identification was performed in the Botany Department, Faculty of Science, Mansoura University, Mansoura (Egypt).

### 4.2. Extraction of Essential Oils

Previously collected *M. spicata* and *M. longifolia* healthy fresh leaves at age one month were prewashed with tap water, disinfected with 2% sodium hypochlorite for 30 min, then rinsed with sterile distilled water. Air-dried plant leaves were homogenized to a fine powder in a mill, stored in airtight dark bottles, and kept until use. Leaf powders were subjected to hydrodistillation for 3 h using a Clevenger-type apparatus, Shiva Scientific Glass Pvt. Ltd., New Delhi, India [72].

### 4.3. GC–MS of Essential Oils

The chemical composition of the volatile content of two studied EOs was determined using GC–MS- QP2010 Ultra analysis system (Shimadzu, Tokyo, Japan). Compounds were separated on an Inc DB-5 60 m × 0.25 mm/0.25 micron column (Agilent Technologies, Santa Clara, CA, USA). The oven temperature program was initiated at 50 °C, held for 3 min, then increased at rate of 8 °C to 250 °C min^−1^ and held for 10 min. The spectrophotometer was operated in electron impact mode. The injector, interface, and ion source were kept at 250 °C, 250 °C, and 220 °C, respectively. Split injection (1 μL diluted sample in n-hexane (1:1, *v*/*v*) injected) was conducted with a split ratio of 1:20 and column flow of 1.5 mL/min, and helium was the carrier gas.

Identification of the components of the sample was based on a comparison of their relative indices and mass spectra by computer matching with WILEY and National Institute of Standards and Technology (NIST08) libraries data (http://webbook.nist.gov accessed on 20 November 2021) provided with the computer controlling GC–MS system. Individual isolated compound identifications were also performed by comparing their mass spectra and retention times with authentic compounds and literature data [73].

### 4.4. Preparation of EOs

The *M. spicata* and *M. longifolia* EOs colloid solutions were prepared by slowly adding 20 mL of *M. spicata* and *M. longifolia* EOs to 1 mL of non-ionic surfactant Tween 80 (1%), and the dispersion was performed under gentle stirring. Then, 80 mL of distilled water was added to reach the final mixture of 100% with continuous stirring using a magnetic stirrer for 30 min. The mixture was fed into a liquefied potato dextrose medium at different concentrations for further in vitro antifungal activity assay and greenhouse experiments.

### 4.5. In Vitro Antifungal Activity of EOs

Assessment of the antifungal activity of *M. spicata* and *M. longifolia* EOs were conducted in vitro and evaluated against *F. oxysporum* radial mycelial growth using the agar plate technique according to Tatsadjieu et al. [74]. The *M. spicata* and *M. longifolia* EOs were liquefied in sterilized PDA media to obtain a final concentration of 0.25%, 0.5%, 0.75%, 1.0% and 1.25%. Twenty mL of broth medium was poured into Petri dishes (90 mm diameter). Plates supplemented with 0.05% of fungicide (nystatin at 0.5 μL/mL) were used as control. Sterile distilled water was used in the bioassays instead of EO as a negative control. All plates were inoculated with mycelial disc (5 mm diameter) of *F. oxysporum* from the PDA plate margins (5–7 days old). Three replicate plates were used for each treatment. Then, the Petri-dishes were incubated at 25 °C and the fungal colony diameter was measured at 7 days.

### 4.6. Preparation of Fungal Suspension

The fungal suspension was prepared as follows: five discs (5 mm diameter) of mycelia agar plugs (7 days old) were added to 1 kg of sterilized maize grains, sand (2:1 *v*/*v*). Ten mL of sterile water was added to the last mixture in a 2 L flask and incubated at 25 ± 2 °C for two weeks. After that, the mixture was put into plastic pots (20 cm diameter). Five sterile discs of PDA medium were inoculated into a control flask [75].

### 4.7. Greenhouse Experiments

Seeds of tomato were surface sterilized in sodium hypochlorite for 30 min, washed five times in sterile water, and germinated in peat moss for three weeks (21DAS). The experiment was irrigated regularly and subsequently moved to experimental pots. Four weeks later, tomato seedlings were removed and their roots were washed and transplanted into the 20 cm diameter pots filled with pasteurized sandy clay soil at 0.9 kg per pot. The seedlings were treated with *M. spicata* and *M. longifolia* EOs at 1.25% and 1.0%, respectively, in the rhizosphere soil. Pots were arranged in a randomized complete block design with three replications. In the first experiment, the pots were divided into two main groups: untreated plants as negative control (C) and plants inoculated with *F. oxysporum* fungal suspension as positive control (P). In the second experiment, after 2 weeks from inoculation, negative control was treated with 50 mL *M. spicata* EOs (1.25%) (T1) and 50 mL *M. longifolia* EOs (1.0%) (T2). In addition, the positive control was treated with 50 mL *M. spicata* EOs (1.25%) (P + T1) and 50 mL *M. longifolia* EOs (1.0%) (P+T2). All the plants continued growth after transplantation with regular irrigation every 3 days for 2 weeks in a greenhouse at 22/16 °C, 65–70% humidity. We then evaluated all pots for the incidence of *F. oxysporum* root rot.

#### 4.7.1. Disease Assessments

The disease severity (DS) was evaluated using the 0–5 scale described by Filion et al. [76]:

Disease severity (%) = (∑ ab/AK) × 100

where a = number of diseased plants with the same infection degree, b = infection degree, A = total number of the evaluated plants, and K = the greatest infection degree.

Whereas the disease incidence (DI) was calculated according to the following equation:

Disease incidence (%) = (a/A) × 100

where a = number of diseased plants, and A = total number of evaluated plants.

#### 4.7.2. Analysis of Plant Growth Parameters

Tomato seedlings of 21-day samples were collected to measure morphological traits. Three plants of each experiment were harvested and transferred to the laboratory and carefully uprooted, washed using tap water for measuring plant height and shoot and root fresh weight. Shoot and root dry weight were measured after oven drying at 40 °C for 48 h.

According to Lichtenthaler et al [77] method, chlorophyll content was determined after 35 days using spectrophotometry. The photosynthetic pigments were ground and extracted from 0.5 g of a third of the fully expanded plant leaves between 8:00 and 10:00 a.m., and suspended in 10 mL of 80% (*v*/*v*) acetone in the dark using a pestle and mortar. Extracts were filtrated and the content of total chlorophyll was determined at 645 nm and 663 nm.

### 4.8. Electrolytes Leakage

Determination of electrolytes leakage was conducted by adding 200 mg of fresh tomato leaves to a test tube containing 4 mL of de-mineralized water and shacked for 30 min. It was then rinsed 3 times to eliminate surface electrolytes [78]. Malondialdehyde (MDA) content was examined in the fresh tomato leaves using the method described by Heath and Packer [79]. Briefly, the MDA contents were determined after centrifugation (12,000× *g*) for 10 min; the absorbance reading was 600 nm and 532 nm using a UV–VIS spectrometer (Jenway, Tokyo, Japan).

### 4.9. Determination of Total Phenolic and Flavonoid Contents

Total phenolic content (TPC) of tomato was measured by dissolving 5 mg of air-dried leaf powder in 10 mL methanol according to Slinkard and Singleton [80] using Folin–Ciocalteu reagent protocol. Total flavonoid content (TFC) of tomato leaves was evaluated using the aluminum chloride colorimetry method described by Chavan et al. [81]. A standard calibration curve was constructed using quercetin in different concentrations (0.05–1 mg/mL). Tomato extract or quercetin (2 mL) was mixed with 500 μL of 10% aluminum chloride solution and 500 μL of 0.1 mM sodium nitrate solution. The absorbance of the reaction mixture was measured after incubation at room temperature for 30 min at wavelength 430 nm using a UV–VIS spectrometer (Jenway, Tokyo, Japan). Soluble protein content (PC) was estimated in both control and treated plants following Bradford [82] using Coomassie Brilliant Blue G-250 dye and the absorbance was recorded at 595 nm using bovine serum albumin as standard.

### 4.10. Assay of Antioxidant Enzymes

Antioxidant enzymes were extracted by homogenizing 1 gm fresh tomato leaf tissue in chilled 50 mM phosphate buffer (pH 7.0) supplemented with 1% polyvinyl pyrolidine and 1 mM EDTA using prechilled pestle and mortar. After centrifuging at 18,000× *g* for 30 min at 40 °C, the supernatant was used for enzyme assay. Determination of the activity of superoxide dismutase (SOD, EC 1.15.1.1) and NBT photochemical reductions was recorded at 560 nm using the Bayer and Fridovich [83] method in a 1.5 mL assay mixture containing sodium phosphate buffer (50 mM, pH 7.5), 100 μL EDTA, L-methionine, 75 μM NBT, riboflavin, and 100 μL enzyme extract.

The catalase assay (CAT, EC1.11.1.6) activity method of Luck [84] was used and monitored the change in absorbance at 240 nm for 2 min. For the calculation, an extinction coefficient of 39.4 mM^−1^ cm^−1^ was used. Ascorbate peroxidase (APX, EC 1.11.1.11) activity was tested by monitoring absorption change at 290 nm for 3 min in a 1 mL reaction mixture containing potassium phosphate buffer (pH 7.0), 0.5 mM ascorbic acid, hydrogen peroxide, and enzyme extract. The calculation of the extinction coefficient of 2.8 mM^−1^ cm^−1^ was used [85].

### 4.11. Gene Expression

According to the manufacturer’s protocol, total mRNA was isolated from 0.5 g tomato plant root of control and all treatments using the Plant RNA Kit (Sigma-Aldrich, St. Louis, MO, USA). The purified RNA was quantitated using SPECTROstar Nano (BMG LABTECH, Ortenberg, Germany). For each sample, 10 μg total RNA was treated with DNAse RNAse-free (Fermentas, Waltham, MA, USA), 5 μg of which was reverse transcribed in a reaction mixture consisting of oligo dT primer (10 pmL/μL), 2.5 μL 5X buffer, 2.5 μL MgCl2, 2.5 μL 2.5 mM dNTPs, 4 μL from oligo (dT), 0.2 μL (5 Unit/μL) reverse transcriptase (Promega, Walldorf, Germany), and 2.5 μL RNA. RT-PCR amplification was performed in a thermal cycler PCR, programmed at 42 °C for 1 h and 72 °C for 20 min. Quantitative real-time PCR was carried out on 1 μL 1:10 diluted cDNA templates by triplicate using the real-time analysis (Rotor-Gene 6000, QIAGEN GmbH, Hilden, Germany) system. The primer sequences used in qRT-PCR are given in Table 4. Primers of three PRs (PR3, PR12) genes, three WRKY transcriptional factors (WRKY1, WRKY4, WRKY33, and WRKY53 TFs genes), and housekeeping gene (reference gene) were used for gene expression analysis using SYBR^®^ Green-based method. The reaction mixture consists of 1 µL of template, 10 µL of SYBR Green Master Mix, 2 µL of reverse primer, 2 µL of forwarding primer, and sterile distilled water for a total reaction volume of 20 µL. PCR assays were performed using the following conditions: 950 °C for 15 min followed by 40 cycles of 950 °C for 30s and 600 °C for 30 s. The CT of each sample was used to calculate ΔCT values (target gene CT subtracted from β-Actin gene CT [86]). The relative gene expression was determined using the 2-ΔΔCt method [87].

### 4.12. Statistical Analyses

All the experiments were performed in triplicates. Obtained data and results were expressed as mean ± standard deviation (±SD). Some experiments were arranged in a completely randomized blocks design and data were statistically analyzed by one-way ANOVA test using SPSS 16. The probability values *p* ≤ 0.05 were considered statistically significant based on Duncan’s least significant difference test. The heatmap was constructed to study the similarity and dissimilarity among studied taxa based on essential and non-essential amino acids using the TBtools package [90].

## 5. Conclusions

We can conclude that EOs of two *Mentha* aromatic plants (*M. spicata* and *M. longifolia*) have the highest potential against Fusarium root rot disease for tomatoes. The chemical essential oils of these EOs have a lethal effect against *F. oxysporum* fungi. In addition, these EOs enhance the growth of tomato plants by increasing their physiological activities. Due to the pathogen attack, this activity could be induced by the plant defense system. This activity was regulated by different families of genes such as WRKY and PR proteins. On the other hand, *L. esculentum* seed priming or seedling root treatments with *M. spicata* and *M. longifolia* EOs could be used at lower concentrations (1.0–1.25%) to enhance seedling growth and alleviate the adverse effects of the fungal disease by supporting antioxidant enzymes and total phenols accumulation which could help make seeds or plants tolerate oxidative stress conditions further. Finally, the EOS of *Mentha* medicinal plants can be used as a safe alternative to fungicides that improve the growth of the infected plant without defects in plant and human health.

## Figures and Tables

**Table 1:** The relative percentage of the area of peak of *M. spicata* EO constituents.

Quantitative ID	Component Identified	Retention Time (min)	Retention Index (RI)	Area (%)	Identification
1	Piperitone	5.20	1245	12.34	RI, MS
2	IR-alpha-pinene	7.82	933	0.76	RI, MS
3	D-limonene	11.83	1031	0.77	RI, MS
4	Cis-P-menthan	17.63	984	0.99	RI, MS
5	Menthone	18.13	1150	0.67	RI, MS
6	P-menthan-3-ol alcohol	18.55	1164	2.27	RI, MS
7	Alpha-terpin	19.49	1187	6.11	RI, MS
8	Gamma-terpineol	19.73	1185	0.82	RI, MS
9	β-caryophyllene	27.19	1325	18.7	RI, MS
10	Butanedioic acid	32.28	1580	6.26	RI, MS
11	Ethyl 4-heptyl ester	36.54	1378	8.83	RI, MS
12	Adipic acid	56.70	1507	13.24	RI, MS
13	Menthol	66.70	1182	12.08	RI, MS
14	Thymol	72.10	1290	14.62	RI, MS
15	Others	-	-	1.54	RI, MS

MS: Mass spectrometry (GC–MS).

**Table 2:** The relative percentage of the area of peak *M. longifolia* EO constituents.

Quantitative ID	Component Identified	Retention Time (min)	Retention Index (RI)	Area (%)	Identification
1	Alpha piene	4.05	933	6.87	RI, MS
2	β-pinene	12.21	964	0.54	RI, MS
3	D-limonene	12.43	1031	10.33	RI, MS
4	Borneol	12.72	1165	0.38	RI, MS
5	β-terpinyl acetate	16.89	1267	0.47	RI, MS
6	Menthol	24.32	1182	44.17	RI, MS
7	Menthone	26.48	1150	0.55	RI, MS
8	Menthyl acetate	29.63	1294	18.36	RI, MS
9	Linalool	31.74	1083	0.43	RI, MS
10	Eugenol	43.6	1209	0.64	RI, MS
11	Carvone	46.89	1242	0.33	RI, MS
12	Thymol	5.08	1290	11.23	RI, MS
13	Cavacrol	59.55	1298	0.36	RI, MS
14	Cis-jasmone	63.41	1394	0.56	RI, MS
15	Cinerolone	63.52	1641	0.53	RI, MS
16	Caryophyllene	64.32	1418	0.74	RI, MS
17	β-farnesene	64.38	1452	0.35	RI, MS
18	β-cubebene	64.57	1389	0.36	RI, MS
19	Alpha-cadinol	64.78	1627	0.82	RI, MS
20	Others	-	-	1.98	RI, MS

**Table 3:** Effects of *M. spicata* and *M. longifolia* EOs on *L. esculentum* seedling growth under Fusarium root rot disease infection.

	EOs Treatment	PH (cm)	SFW (g)	SDW (g)	RFW (g)	RDW (g)
Control	Control Plants	24.32 ± 0.02 c	15.44 ± 0.02 c	1.98 ± 0.01 c	1.52 ± 0.01 c	0.16 ± 0.01 c
	1.25% *M. spicata*	28.9 ± 0.01 b	15.96 ± 0.02 b	2.09 ± 0.01 b	1.64 ± 0.01b	0.18 ± 0.01 b
	1% *M. longifolia*	32.42 ± 0.02 a	16.33 ± 0.01 a	2.34 ± 0.01 a	1.87 ± 0.01 a	0.24 ± 0.01 a
Pathogen	Infected Plants	16.54 ± 0.02 f	9.52 ± 0.03 f	0.97 ± 0.01 f	1.21 ± 0.02 f	0.1 ± 0.01 f
	1.25% *M. spicata*	18.76 ± 0.01 e	12.97 ± 0.01 e	1.67 ± 0.01 e	1.34 ± 0.15 e	0.12 ± 0.01 e
	1% *M. longifolia*	19.86 ± 0.02 d	13.64 ± 0.01 d	1.85 ± 0.01 d	1.41 ± 0.02 d	0.14 ± 0.01 d

PH = plant height, SFW = shoot fresh weight, SDW = shoot dry weight, RFW = root fresh weight, RDW = root dry weight, and EOs = essential oils. Different letters indicate significant differences between different treatments at *p* ≤ 0.05. Data are expressed as the mean of three replicates ± SDs.

**Table 4:** Sequences of primers used in qRT-PCR analysis.

Gene Name		Sequence	ID & Reference
Chitinase (PR3)	F	5′- ATGGCGGAAACTGTCCTAGTGGAA -3′	Medeiros et al. [88]
	R	5′ ACATGGTCTACCATCAGCTTGCCA -3′	
Defensin (PR12)	F	5′- TCACCAAACTATTGGATTTCAA -3′	Hafez et al. [89]
	R	5′- GACTCAATTTTTGACTTCTTAATCC -3′	
WRKY1	F	5′- CGCAACTCAAAGAGACGGAAG-3′	Solyc07g047960.2.1
	R	5′- CATTGACTACATCCACTTCACTGC-3′	
WRKY4	F	5’- CGTTGCACATACCCTGGATG -3′	Solyc05g012770.2.1
	R	5′- GGCCTCCAAGTTGCAATCTC -3′	
WRKY33	R	5′- CCACCTCCTTCACTTCCATT -3′	Solyc09g014990.2.1
	F	5′- GATGGAAAACTCCCAGTCGT -3′	
WRKY53	F	5′- CACATACCGAGGCTCCCATAA -3′	Solyc08g008280.2.1
	R	5′- CCTGTTGGATAAACGGCTTGG -3′	
β-Actin	F	5′- TCCTTCTTGGGTATGGAATCCT-3′	NM_007393.5
	R	5′- CAGCACTGTGTTGGCATAGA-3′	

## Data Availability

Relevant data applicable to this research are within the paper.

## Supplementary Materials

The following supporting information can be downloaded at: https://www.mdpi.com/article/10.3390/plants11020189/s1, Figure S1. Relative expression level of WRKY1 gene in *S. lycopersicum* seedling under *Fusarium* inoculation and application of *M. spicata* and *M. longifolia* EOs. Different letters indicate significant differences between different treatments at *p* ≤ 0.05. Figure S2. Relative expression level of WRKY4 gene in *S. lycopersicum* seedling under *Fusarium* inoculation and application of *M. spicata* and *M. longifolia* EOs. Different letters indicate significant differences between different treatments at *p* ≤ 0.05. Figure S3. Relative expression level of WRKY33 gene in *S. lycopersicum* seedling under *Fusarium* inoculation and application of *M. spicata* and *M. longifolia* EOs. Different letters indicate significant differences between different treatments at *p* ≤ 0.05. Figure S4. Relative expression level of WRKY53 gene in *S. lycopersicum* seedling under *Fusarium* inoculation and application of *M. spicata* and *M. longifolia* EOs. Different letters indicate significant differences between different treatments at *p* ≤ 0.05. Figure S5. Relative expression level of Chitinase gene in *S. lycopersicum* seedling under *Fusarium* inoculation and application of *M. spicata* and *M. longifolia* EOs. Different letters indicate significant differences between different treatments at *p* ≤ 0.05. Figure S6. Relative expression level of defensin gene in *S. lycopersicum* seedling under *Fusarium* inoculation and application of *M. spicata* and *M. longifolia* EOs. Different letters indicate significant differences between different treatments at *p* ≤ 0.05.
