# Supplementary material for: Biochemical Characterization, Antifungal Activity, and Relative Gene Expression of Two Mentha Essential Oils Controlling Fusarium oxysporum, the Causal Agent of Lycopersicon esculentum Root Rot
Source: Plants (Basel). 2022 Jan 11;11(2):189. doi: 10.3390/plants11020189 (PMC8781374; doi:10.3390/plants11020189)
Supplement: Supplementary file 1 [file plants-11-00189-s001.zip › plants-1531612-supplementary.pdf]

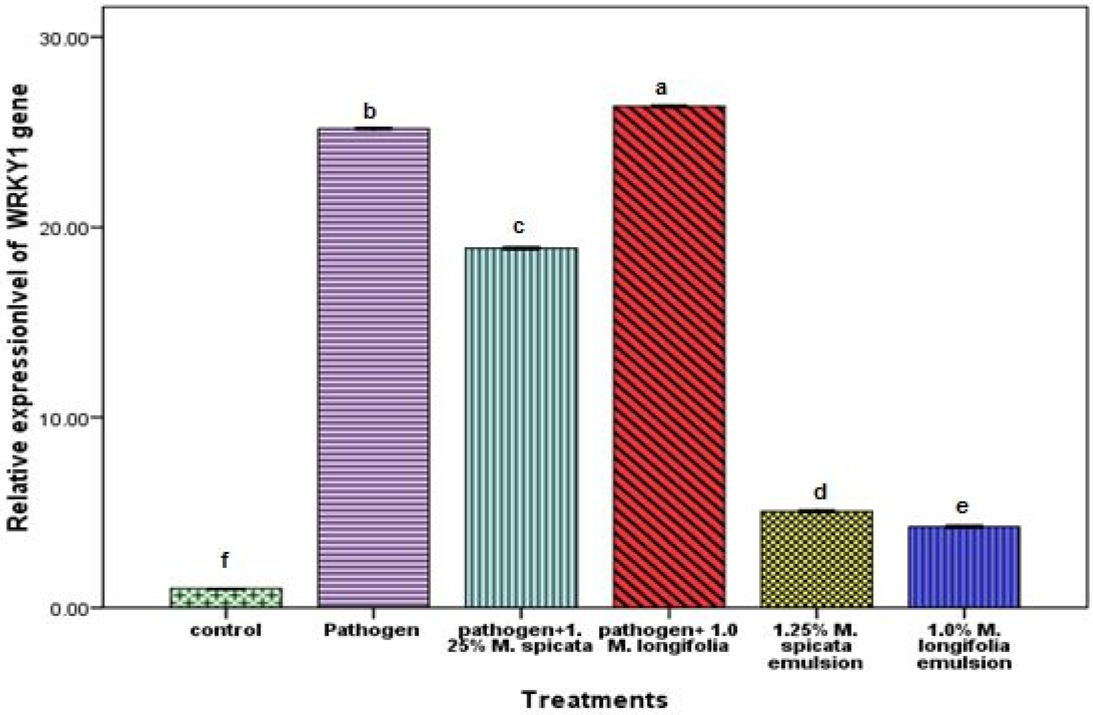

Figure S1. Relative expression level of WRKY1 gene in *S. lycopersicum* seedling under *Fusarium* inoculation and application of *M. spicata* and *M. longifolia* EOs. Different letters indicate significant differences between different treatments at $p \leq 0.05$.

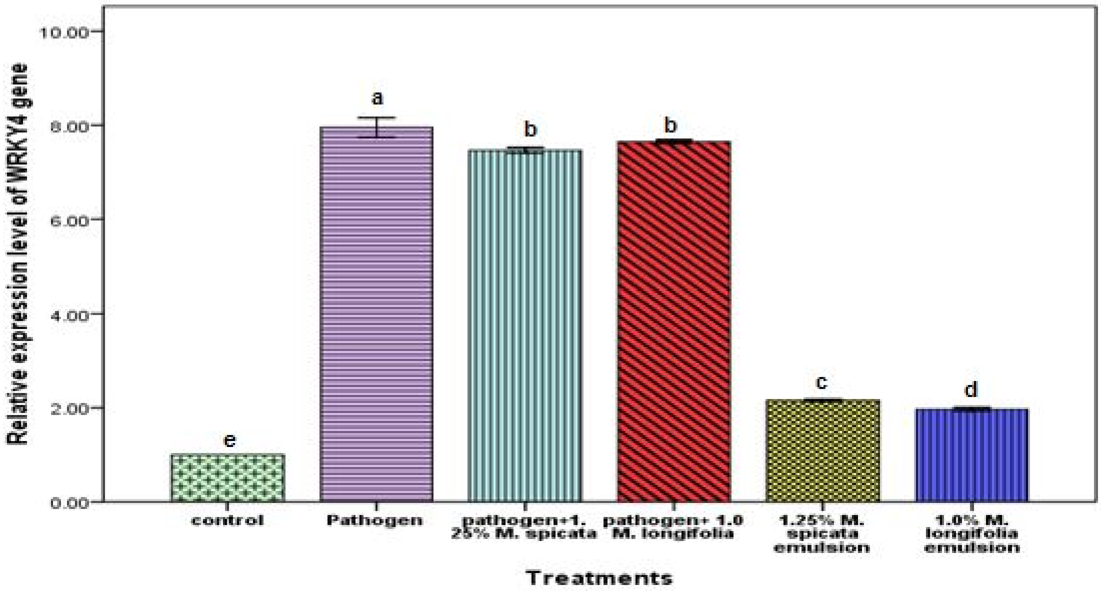

Figure S2. Relative expression level of WRKY4 gene in *S. lycopersicum* seedling under *Fusarium* inoculation and application of *M. spicata* and *M. longifolia* EOs. Different letters indicate significant differences between different treatments at $p \leq 0.05$.

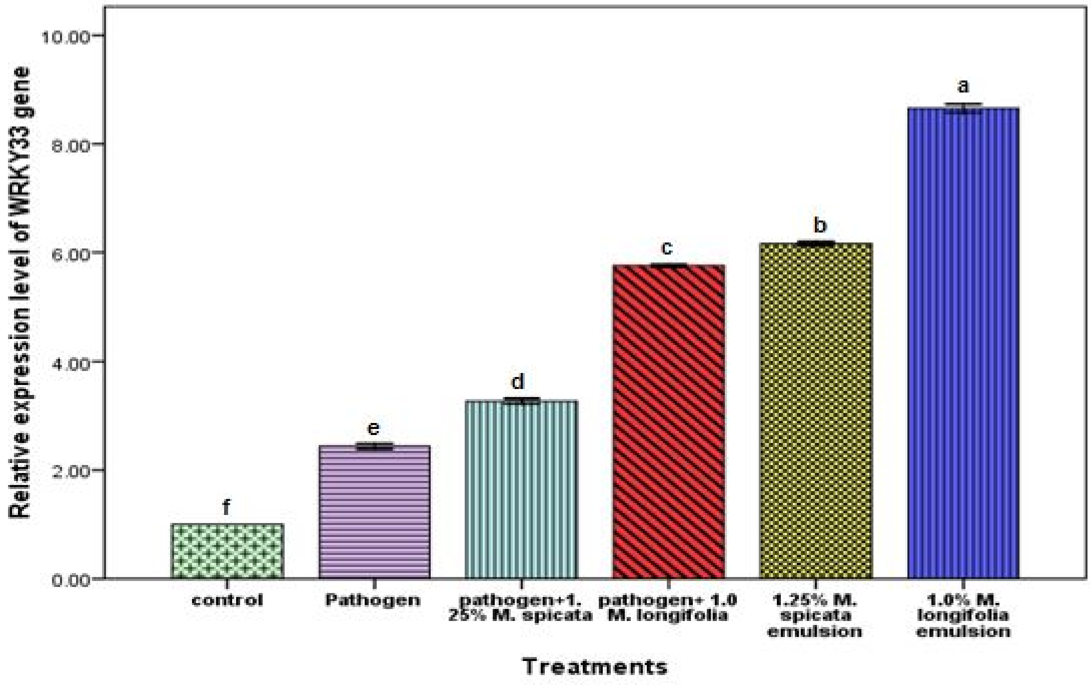

Figure S3. Relative expression level of WRKY33 gene in *S. lycopersicum* seedling under *Fusarium* inoculation and application of *M. spicata* and *M. longifolia* EOs. Different letters indicate significant differences between different treatments at $p \leq 0.05$.

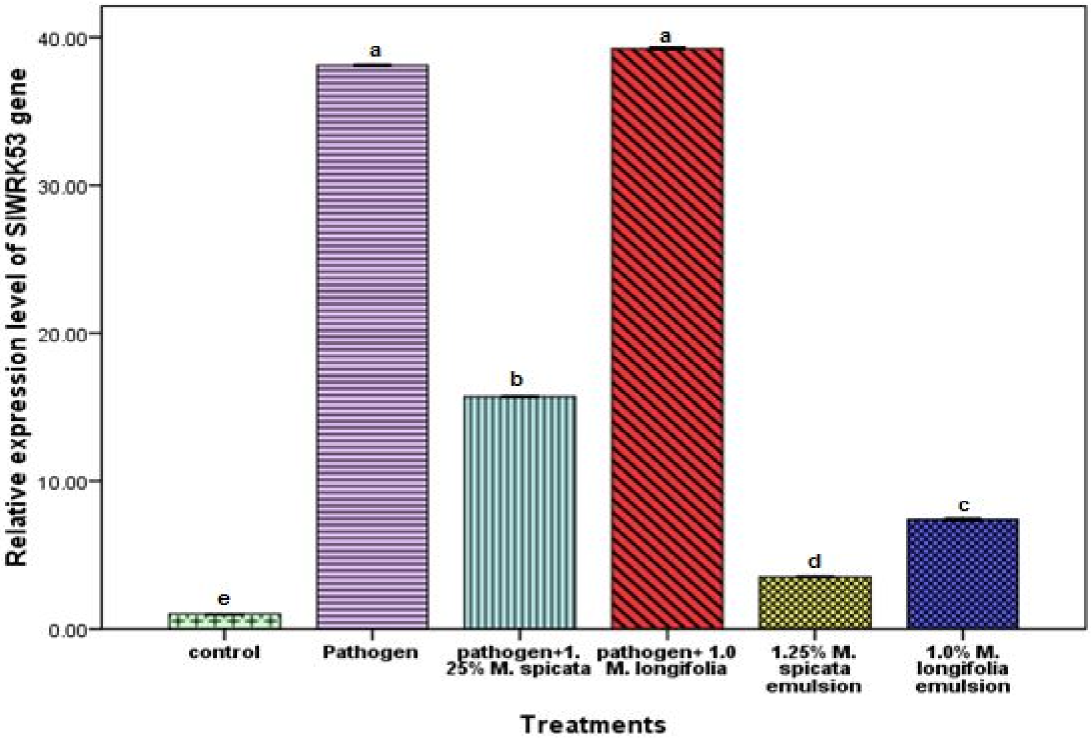

Figure S4. Relative expression level of WRKY53 gene in *S. lycopersicum* seedling under *Fusarium* inoculation and application of *M. spicata* and *M. longifolia* EOs. Different letters indicate significant differences between different treatments at $p \leq 0.05$.

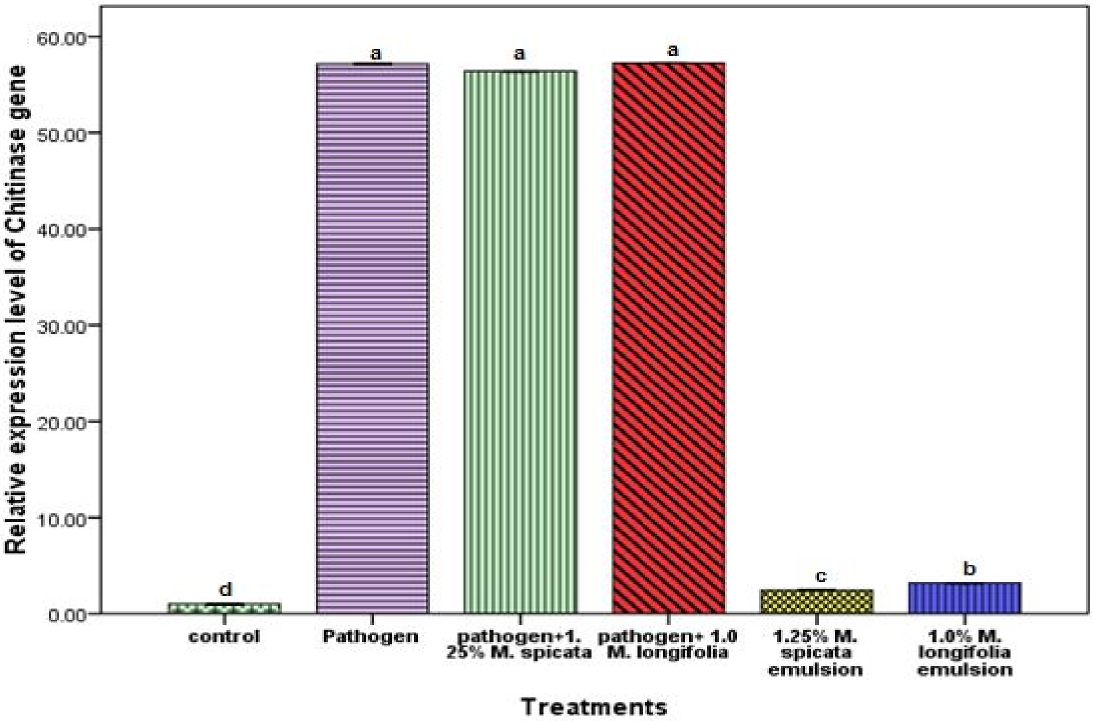

Figure S5. Relative expression level of Chitinase gene in *S. lycopersicum* seedling under *Fusarium* inoculation and application of *M. spicata* and *M. longifolia* EOs. Different letters indicate significant differences between different treatments at p ≤ 0.05.

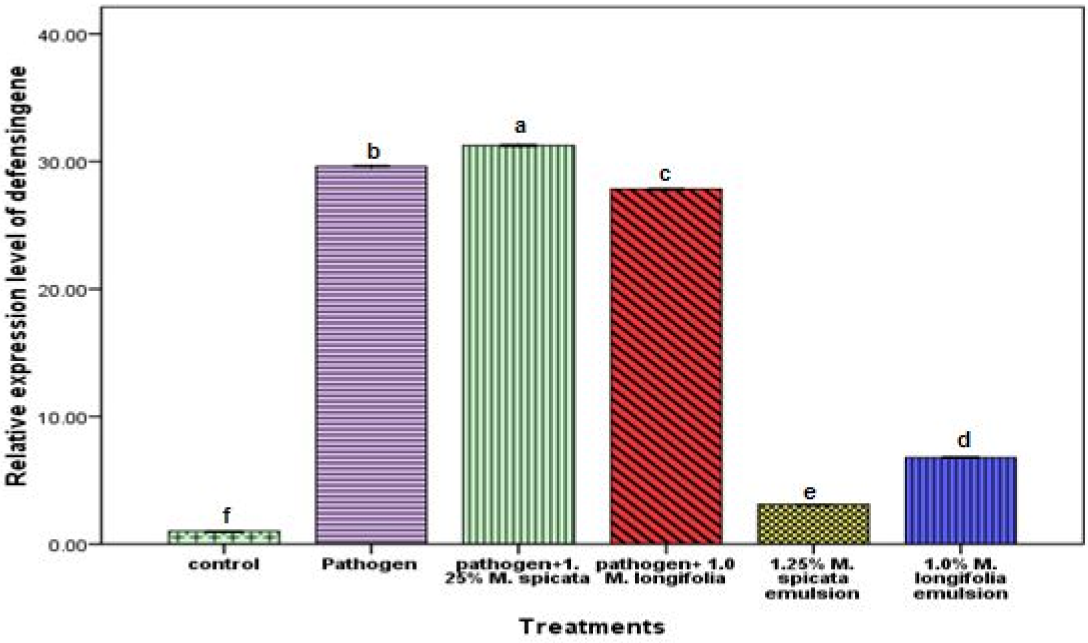

Figure S6. Relative expression level of defensin gene in *S. lycopersicum* seedling under *Fusarium* inoculation and application of *M. spicata* and *M. longifolia* EOs. Different letters indicate significant differences between different treatments at p ≤ 0.05.
